# Decoding nucleoside supplementation: how thymidine outperforms ribonucleosides in accelerating mammalian replication forks

**DOI:** 10.1093/nar/gkaf1035

**Published:** 2025-10-14

**Authors:** Praveen Pandey, Kiminori Kurashima, Göran Bylund, Erik Johansson, Tomomi Tsubouchi, Andrei Chabes

**Affiliations:** Department of Medical Biochemistry and Biophysics, Umeå University, Umeå SE-901 87, Sweden; Laboratory of Stem Cell Biology, National Institute for Basic Biology, National Institutes of Natural Sciences, 38 Nishigonaka, Myodaiji, Okazaki Aichi 444-8585, Japan; Department of Medical Biochemistry and Biophysics, Umeå University, Umeå SE-901 87, Sweden; Department of Medical Biochemistry and Biophysics, Umeå University, Umeå SE-901 87, Sweden; Laboratory of Stem Cell Biology, National Institute for Basic Biology, National Institutes of Natural Sciences, 38 Nishigonaka, Myodaiji, Okazaki Aichi 444-8585, Japan; Department of Medical Biochemistry and Biophysics, Umeå University, Umeå SE-901 87, Sweden

## Abstract

Disruptions in deoxynucleoside triphosphate (dNTP) supply impair DNA replication and lead to genomic instability. While exogenous ribonucleosides (rNuc) have been suggested to alleviate replication stress by increasing dNTP levels, their precise metabolic effects remain unclear. Here, we show that rNuc supplementation primarily elevates CTP and UTP levels, with only modest increases in dCTP, and has minimal impact on replication fork speed across multiple mammalian cell lines. In contrast, thymidine (dThd), either alone or in combination with rNuc—as in EmbryoMax Nucleosides—significantly increases dTTP and dGTP levels, leading to accelerated replication fork progression. Notably, dThd, rather than rNuc, drives fork acceleration and counteracts fork slowdown caused by elevated dUTP, consistent with primer extension assays showing that dUTP transiently inhibits Pol ϵ-mediated DNA synthesis at template adenines. These results clarify the distinct roles of nucleosides in nucleotide metabolism, providing a mechanistic basis for how dThd promotes fork progression and preserves genomic stability.

## Graphical Abstract

**Graphical Abstract:**
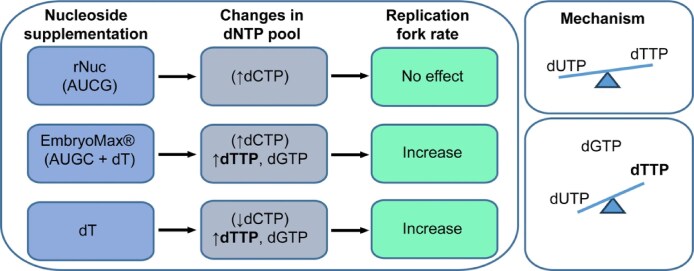

## Introduction

Exogenously supplied deoxyribonucleosides (dNuc), precursors of deoxyribonucleotide triphosphates (dNTPs), are salvaged by mammalian cells and help alleviate replication stress or mitigate dNTP insufficiency [1–4]. Interestingly, ribonucleosides (rNuc)—the direct precursors of ribonucleotide triphosphates (rNTPs)—can also serve this function. For example, Bester *et al.* reported that exogenously supplied rNuc—adenosine (Ado), uridine (Urd), cytidine (Cyd), and guanosine (Guo), each at a concentration of 50 μM—restored replication dynamics in cells with decreased dNTP pools caused by the expression of HPV-16 E6 and E7 proteins [5]. Similarly, Kotsantis *et al.* observed that 10 μM rNuc improved replication fork speed and reduced 53BP1 foci formation induced by HRAS^V12^, despite no observed reduction in dNTP or rNTP levels in these cells [6]. Additionally, Burrell *et al.* found that 0.3 μM or 30 μM rNuc reduced replication-stress-associated DNA damage and chromosome missegregation events in CIN^+^ cells [7]. Aird *et al.* further demonstrated that the exogenous rNuc concentrations as low as 50 nM were sufficient to overcome RAS oncogene-induced senescence and rescue aberrant replication caused by decreased dNTP levels [8].

Exogenous rNuc are often provided premixed, as for example in the commercially available 100× EmbryoMax Nucleoside (EM Nuc) solution, which contains 3 mM of each rNuc (Supplementary Table S1). Notably, this formulation also includes one deoxyribonucleoside (dNuc), 1 mM thymidine (dThd). Supplementation with EM Nuc at 1× (yielding 30 μM rNuc and 10 μM dThd) has been reported to alleviate replication stress in hematopoietic and progenitor cells lacking the single-stranded DNA binding proteins SSB1 and SSB2 [9], to accelerate replication fork progression in Cdh1-deficient cells [10], and to rescue the reduced replication fork speed observed upon expression of reprogramming factors [11]. Furthermore, a 2× supplementation of EM Nuc was shown to increase replication fork speed and reduce double-strand DNA breaks in cells with inhibited Wee1 kinase [12], as well as to accelerate replication fork speed throughout the S phase in ES cells and during early S phase in MEFs [13]. Lower concentrations also provided beneficial effects: EM Nuc supplementation at 0.5× reduced replication stress, DNA damage, and aberrant mitosis in human pluripotent cells [14], while a 0.1× concentration increased mitotic DNA synthesis, prevented the accumulation of mitotic γH2AX foci, anaphase aberrations, and micronuclei, and decreased chromosomal instability in Chk1-depleted cells [15]. As such, EM Nuc has become a standard component of media for culturing embryonic and induced pluripotent stem cells.

The mechanism by which exogenous rNuc supplementation alleviates replication stress is unclear. One possibility is that rNuc are converted into dNTPs, thereby elevating their levels. After being transported into the cells, rNuc are phosphorylated by nucleoside kinases to ribonucleoside monophosphates (rNMPs), and then further phosphorylated to ribonucleoside diphosphates (rNDPs) and ultimately to rNTPs. Since ribonucleotide reductase (RNR) converts rNDPs to deoxyribonucleoside diphosphates (dNDPs), which are then phosphorylated to form dNTPs (Supplementary Fig. S1), an increase in rNMP–rNDP–rNTP pools via exogenous rNuc supplementation could, in principle, stimulate dNTP synthesis.

Another possibility is that the dThd included in the EM Nuc formulation, rather than the rNuc themselves, is responsible for alleviating replication stress. dThd is phosphorylated to dTMP by thymidine kinases, then to dTDP by thymidylate kinase, and finally to dTTP by nucleoside diphosphate kinases. Given that the intracellular concentration of dTTP is in the low micromolar range [16], supplementation with 10 μM dThd (as in 1× EM Nuc) could significantly increase dTTP levels, thereby altering the balance of dNTP pools.

Despite the widespread use of nucleosides to alleviate replication stress, the specific effects of different combinations of exogenous nucleosides on rNTP and dNTP pools remain poorly understood. In this study, we investigate how exogenously supplied rNuc, rNuc combined with dThd (EM Nuc), and dThd alone affect rNTP and dNTP pools as well as replication fork speed in several commonly used mammalian cell lines, interpreting our results in the context of current knowledge about mammalian nucleotide metabolism.

## Materials and methods

### Ethical compliance

All animal experiments were conducted in accordance with Directive 2010/63/EU and approved by the Swedish Research Animal Welfare Agency under ethical permit A4-2021 (Umeå Regionala Djurförsöksetiska nämnd). Mice were housed under standard conditions with a 12-h light/dark cycle and provided ad libitum access to food and water.

### Reagents

Ado (A4036), Guo (G6752), Urd (U3750), Cyd (C4654), dAdo (D7400), dGuo (D7145), dCyd (D3897), and dThd (T1895) from Sigma–Aldrich were dissolved in water. EmbryoMax^®^ Nucleosides (100×) (ES-008-D, Sigma–Aldrich) containing 0.73 g/L Cyd, 0.85 g/L Guo, 0.73 g/L Urd, 0.8 g/L Ado, and 0.24 g/L dThd were added directly to the cell culture media. Unless otherwise stated, the indicated concentrations of rNuc or dNuc mixes, for example 50 μM, refer to 50 μM of each nucleoside species in the mix. Nucleosides were supplemented to logarithmically growing cells in 15 cm culture dishes for nucleotide measurement and in six-well plates for the DNA fiber assay. Acronyms and abbreviations used throughout are summarized in Supplementary Table S1.

### Biological resources

All cell lines were cultured in growth media as recommended by the American Type Culture Collection. Media were supplemented with fetal bovine serum (FBS) (10500064, Gibco) and antibiotics 100 μg/ml streptomycin and 100 units/ml penicillin (15140122, Gibco). Cells were grown as a monolayer in a cell culture incubator maintained at 37°C with a humidified atmosphere containing 5% CO_2_. All cell lines were routinely tested for mycoplasma contamination and confirmed to be mycoplasma-free throughout the course of this study. Specific culture conditions for each cell line are as follows:

BJ Human Diploid Foreskin Normal Fibroblast Cells (CRL-2522), BALB/3T3 Mouse Fibroblast Cells (CCL-163), A549 Human Lung Cancer Cells (CCL-185), HeLa Human Cervical Cancer Cells (CCL-2), and U2OS Human Bone Osteosarcoma Cells (HTB-96): Cultured in Dulbecco’s modified Eagle’s medium (DMEM) (61965026, Gibco) supplemented with 10% FBS.

Mouse embryonic fibroblast cells (MEFs), used in the experiments shown in Fig. 2, were isolated from E11.5 embryos of C57BL/6 mice using the Pierce Mouse Embryonic Fibroblast Isolation Kit (88279, Thermo Fisher Scientific), following the manufacturer’s instructions, and immortalized with the SV40 large T antigen using the pBSSVD2005 plasmid (Addgene), as described in the protocol available at https://media.addgene.org/data/45/42/165f51de-af64-11e0-90fe-003048dd6500.pdf. The cells were cultured in DMEM supplemented with 10% FBS and 1× MEM Non-Essential Amino Acids (11140-050, Gibco).

Mouse Embryonic Fibroblast cells SNL [17], used in experiments shown in Fig. 3, were cultured in DMEM (D6546, Sigma) with 10% FBS, 1× NEAA, 2 mM L-Glutamine (WAKO), and 0.1 mM β-mercaptoethanol.

Vero African Green Monkey Kidney Normal Cells (CCL-81): Cultured in DMEM supplemented with 5% FBS.

HT29 Human Colon Cancer Cells (HTB-38) and HCT116 Human Colon Cancer Cells (CCL-247): Cultured in McCoy’s 5A media (M8403, Sigma–Aldrich) supplemented with 10% FBS and 2 mM L-glutamine.

### Nucleotide extraction and measurement

Nucleotides were extracted from cells grown to ∼70% confluence on a single 15 cm dish, except for MEFs and BALB/3T3 cells, which required two plates, and BJ cells, which required three plates per measurement. Media was removed, and cells were washed twice with ice-cold saline (0.9% NaCl) and placed on ice. Cells were then immediately harvested in 450 μl of ice-cold TCA solution (15% TCA and 30 mM MgCl_2_) using a cell scraper, collected in a 1.5 ml Eppendorf tube, snap frozen in liquid nitrogen, and stored at −80°C until further processing.

Samples were processed with modifications as described previously [18]. Briefly, samples were thawed on ice, vortexed for 10 min in a cold room on an Intelli-Mixer™ RM-2 at 99 rpm, and centrifuged at 20 000 × *g* for 2 min at 4°C. The supernatant was extracted twice with 1.4× the volume of dichloromethane-trioctylamine (78% v/v–22% v/v, respectively) by vortexing for 30 s and centrifugation at 20 000 × *g* for 1 min. The upper neutral phase was transferred to a new tube and further purified using an Oasis weak anion exchange (WAX) column (Oasis WAX 3cc cartridge, 186002492, Waters). The WAX columns were sequentially equilibrated by 2 ml of 100% methanol, 2 ml of deionized water, and 2 ml of 50 mM ammonium acetate (pH 4.5). Samples were diluted to 4 ml in 50 mM ammonium acetate (pH 4.5) and passed through the columns. After washing with 2 ml of 50 mM ammonium acetate (pH 4.5) and 2 ml of 0.5% ammonia solution in methanol (v/v), the bound nucleotides were eluted with 1.5 ml of methanol/water/ammonia solution (80%/15%/5%, v/v/v) into glass tubes. Samples were then evaporated to dryness in a Genevac™ miVac Centrifugal Concentrator (MST-23050-B00) for ∼2 h at ∼30°C. Dried samples were dissolved in 200 μl of deionized water and stored at –20°C until HPLC analysis, following a modified method [19]. Briefly, samples were isocratically separated on a 4.6 mm i.d. × 150 mm SunShell C18-WP, 2.6 μm HPLC column (CW6471, ChromaNik Technologies Inc, Japan) using a Shimadzu Nexera series HPLC. The mobile phase contained 0.7 g/L tetrabutylammonium bromide (TBA-Br) (426288, Sigma), 9.2 g/L KH_2_PO_4_ (153184U, VWR Chemicals), and 5.8% (v/v) acetonitrile, pH 5.6 adjusted with 4 M KOH. Before loading, 10 μl of samples were diluted in sample loading buffer. To prepare 10× sample loading buffer, buffer A (11.5 g KH_2_PO_4_ and 0.6375 ml 4 M KOH in 100 ml water) was mixed with buffer B (1.4 g TBA-Br in 100 ml water) in a 1:1 ratio. dNTPs and rNTPs were quantified at 260 nm by comparing peak heights to a standard nucleotide solution.

### DNA fiber assay

The DNA fiber assay was carried out as described previously [13]. Briefly, cells were sequentially labelled with 20 μM IdUrd (I7125, Sigma–Aldrich) and 200 μM CldUrd (C6891, Sigma–Aldrich) for 15 min each. Cells were then washed with medium and resuspended in a fixative solution (3:1 methanol/acetic acid). To stretch DNA fibers on slides, fixed cells were spotted onto aminopropylsilane-coated glass slides. After drying, slides were immersed in a lysis solution [200 mM Tris–HCl, pH 7.5, 50 mM ethylenediaminetetraacetic acid (EDTA) and 0.5% sodium dodecyl sulfate] for 15 min at 37°C. The DNA fibers released from the cells were extended by tilting the slides in a high-humidity chamber for 30 min, then immersed in fixative solution for 2 min, and washed in distilled water. To denature the DNA fibers, the slides were immersed in 2.5 M HCl for 80 min and washed three times in phosphate-buffered saline (PBS). After blocking with PBS containing 1% bovine serum albumin (BSA) for 20 min, slides were incubated for 2 h at room temperature (RT) with anti-IdU (1/400, Sigma–Aldrich, SAB3701448) and anti-CldU (1/100, Abcam, ab6326) antibodies in CanGetSignal A (TOYOBO, NKB-501) to label nascent DNA. Following three washes with PBS containing 0.05% w/v Tween^®^ 20 (PBST), the slides were fixed with 3.7% formaldehyde in PBST for 10 min, washed three times with PBST, and incubated at RT for 1 h with anti-rat IgG conjugated with Alexa Fluor 594 (712-585-153, Jackson ImmunoResearch) and anti-mouse IgG conjugated with Alexa Fluor 488 (115-545-003, Jackson ImmunoResearch) in CanGetSignal A. After three additional washes in PBST, slides were mounted with Vectashield Plus (H-1900, Vector Laboratories). Images were captured using a fluorescence microscope (Olympus) and analyzed using ImageJ software (NIH). To measure fork speed, DNA fibers displaying ∼1:1 length ratio of IdU:CldU were used. The length of the labeled regions was measured, converted to kilobases using 1 μm = 3.5 kb [20], and divided by the 30 min pulse labeling time to calculate fork speed (kb/min), with at least 150 forks examined per sample.

### Human *POLE1* cloning

A codon-optimized DNA sequence encoding the catalytic fragment of human *POLE1* (human isoform B, corresponding to amino acids 1–1174), with flanking restriction sites for NdeI and XhoI, was synthesized and cloned into the vector pEX-A258 (MWG Eurofins Genomics). This construct was then used to subclone the *POLE1* catalytic fragment into an NdeI- and XhoI-digested pET28 expression vector, placing it downstream of a His-tag sequence (Invitrogen). The resulting pET28-*POLE1* plasmid was transformed into *Escherichia coli* strain BL21(DE3) for protein expression.

### Human POLE1 expression and purification

Fifty milliliters of LB medium supplemented with 50 μg/ml kanamycin was inoculated with a single colony of freshly transformed BL21(DE3)/pET28-*POLE1* and grown overnight at 37°C (starter culture). 0.5 ml of the starter culture was used to inoculate eight pre-warmed bottles in an Epiphyte3 bioreactor (Canada), each containing 1.5 liters of terrific broth supplemented with 50 μg/ml kanamycin and 8–10 drops of antifoam (Merck). Growth continued at 37°C until OD_600_ reached 0.9, at which point the cultures were chilled in ice water, and the bioreactor temperature was lowered to 16°C. Once 16°C was reached, protein expression was induced by the addition of IPTG to a final concentration of 0.4 mM. Protein expression proceeded overnight for 16–18 h, followed by cell harvest. Cells were frozen in liquid nitrogen and stored at −80°C until use.

All purification steps were performed at 4°C or on ice. To 60 grams of cells, 130 ml of lysis buffer [25 mM Tris-Acetate (pH 7.8), 300 mM Na-Acetate, 10% glycerol, 5 mM 2-mercaptoethanol, 20 mM MgCl2, 1 mM AEBSF (Merck), one Roche protease inhibitor cocktail tablet, 0.15 g lysozyme (Merck), and 1.5 mg DNase I from bovine pancreas (Merck)] was added. Cells were thawed and resuspended in the buffer by stirring and pipetting. After complete resuspension, the mixture was incubated with lysozyme for 45 min with occasional stirring. Lysozyme treatment was followed by sonication in ice water (5 s on, 5 s off for 2 min, followed by 2 min of cooling, repeated four times). Cell debris was pelleted by centrifugation at 40 000 rpm in a Beckman 45Ti rotor for 1 h. Imidazole was added to the supernatant to a final concentration of 20 mM, and the sample was filtered through a 0.2 μm filter before being loaded onto a 1 ml HisTrap column (Cytiva), equilibrated in Buffer A_300_ [25 mM Tris-Acetate (pH 7.8), 10% glycerol, 300 mM Na-Acetate, 5 mM 2-mercaptoethanol, 20 mM imidazole]. The column was washed sequentially with 20 ml Buffer A_800_ (same as Buffer A_300_ but with 800 mM Na-Acetate), 20 ml Buffer A_800_ supplemented with 50 mM imidazole, and 20 ml Buffer A_300_. Protein was eluted using Buffer A_300_ supplemented with 300 mM imidazole. Protein-containing fractions were pooled, centrifuged at 21 300 × *g* for 15 min, and loaded onto a 1 ml MonoQ column (Cytiva) equilibrated in a buffer containing 25 mM Tris-Acetate (pH 7.8), 10% glycerol, 5 mM 2-mercaptoethanol, and 200 mM Na-Acetate. Elution was performed using a 20 ml linear gradient from 200 to 1000 mM Na-Acetate. hPOLE1-containing fractions were pooled and applied to a 24 ml Superdex 200 Increase column (Cytiva), equilibrated in 25 mM Tris-Acetate (pH 7.8), 10% glycerol, 5 mM 2-mercaptoethanol, and 400 mM Na-Acetate.

The final protein fractions were pooled and diluted to 90 ng/μl with Buffer B_400_ [25 mM Hepes (pH 7.6), 10% glycerol, 400 mM Na-Acetate, 0.05% NP-40, 1 mM DTT, 2 μM Pepstatin A, 2 μM Leupeptin], aliquoted, and stored frozen at −80°C until use.

### Primer extension assay

The Tetrachlorofluorescein (TET)-labeled 50-mer: TET-GATCAGACTGTCCTTAGAGGATACTCGCTCGCAGCCGTCCACTCAACTCA-3′ was annealed to the 80-mer: 3′-CTAGTCTGACAGGAATCTCCTATGAGCGAGCGTCGGCAGGTGAGTTGAGTCGGTCTTGAAAAAGTGACTGATAGTTCGAC-5′ and used as a DNA template for primer extension assays.

Primer extension reactions were initiated by mixing 10 μl of solution A [640 fmol DNA template, 8 mM Mg-Acetate, 125 mM Na-Acetate, 40 mM Tris–HCl (pH 7.8), 1 mM DTT, 100 μg/ml BSA, 8 μM dCTP, 8 μM dATP, 4 μM dGTP, 16 μM dTTP, and 0–2000 μM dTTP or dUTP, as indicated] with 10 μl of solution B [8 mM Mg-Acetate, 125 mM Na-Acetate, 40 mM Tris–HCl (pH 7.8), 1 mM DTT, 100 μg/ml BSA, and 128 fmol hPOLE1]. The 20 μl reactions were incubated at 37°C for 2 min before stopping with an equal volume of stop solution (96% formamide, 20 mM EDTA, bromophenol blue). Reactions were heated at 90°C for 15 min, chilled on ice, and analyzed on a 10% sequencing gel. Gels were scanned using a Typhoon imaging system (Cytiva).

### Statistics and data analysis

All statistical analyses were performed using GraphPad Prism software. Depending on the dataset, statistical significance was determined using one-way ANOVA, two-way ANOVA, or the Mann–Whitney test, as specified in each figure legend. For ANOVA analyses, Bonferroni post hoc multiple comparisons were applied. Statistical significance is indicated in the figures as follows: *P < .05, **P < .01, ***P < .001, ****P < .0001. No asterisk indicates *P* ≥ .05. Error bars represent the standard deviation (SD) from three independent experiments unless otherwise stated.

## Results

### rNuc supplementation elevates CTP, UTP, and dCTP pools

**Figure 1.**
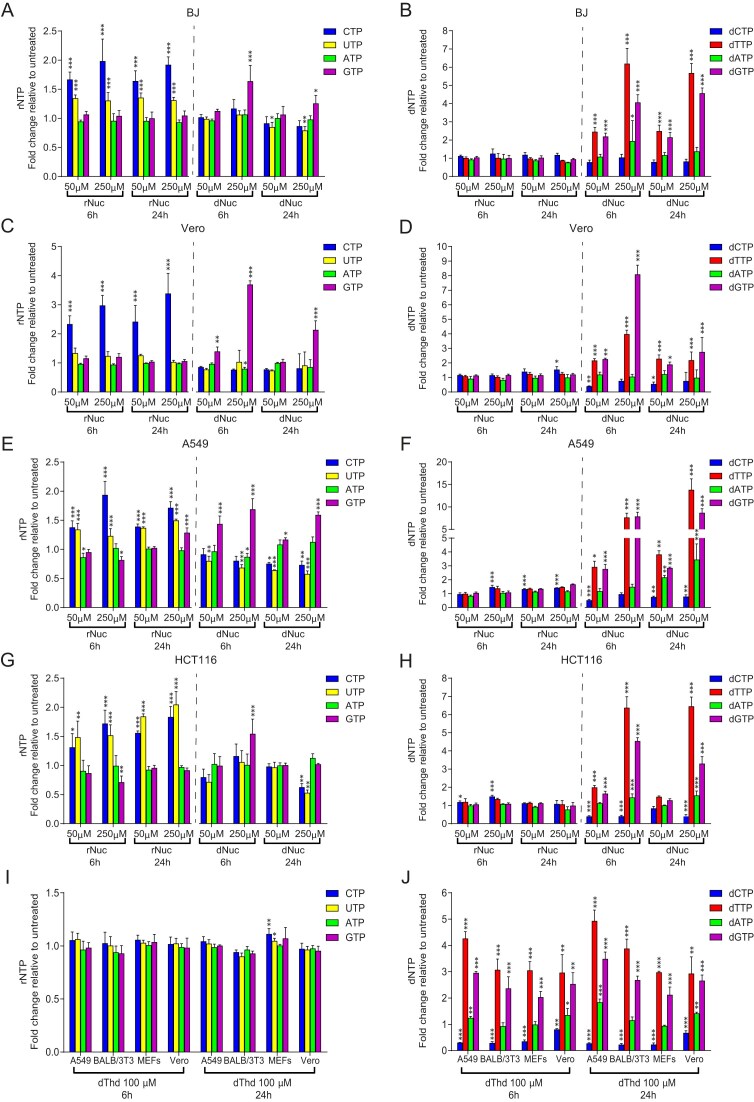
Changes in dNTP and rNTP pools in mammalian cells following rNuc or dNuc supplementation. (**A**, **C**, **E**, **G**) rNTP levels in BJ, Vero, A549, and HCT116 cells after 6 and 24 h of treatment with rNuc (50 μM or 250 μM) or dNuc (50 μM or 250 μM), shown relative to untreated controls. (**B**, **D**, **F**, **H**) dNTP levels in BJ, Vero, A549, and HCT116 cells after 6 and 24 h of treatment with rNuc (50 μM or 250 μM) or dNuc (50 μM or 250 μM), shown relative to untreated controls. (**I**) rNTP levels in A549, BALB/3T3, MEF, and Vero cells after 6 and 24 h of treatment with dThd (100 μM), shown relative to untreated controls. (**J**) dNTP levels in A549, BALB/3T3, MEF, and Vero cells after 6 and 24 h of treatment with dThd (100 μM), shown relative to untreated controls. Error bars represent the SD from three independent experiments. Statistical significance: *P < .05, **P < .01, ***P < .001. No asterisk indicates *P* ≥ .05. Statistical significance was determined using two-way ANOVA followed by Bonferroni post hoc multiple comparisons.

Previous studies with rNuc supplementation have utilized equimolar mixtures, despite the strong intracellular asymmetry observed in both dNTP and rNTP pools [16] and Supplementary Fig. S2. Here, we supplemented six cell lines—BJ, Vero, A549, HCT116, U2OS, and HT29—with 50 μM and 250 μM rNuc mixtures and measured rNTP and dNTP levels after 6 and 24 h. CTP levels increased across all cell lines, while UTP levels increased in some but not all cell lines (Fig. 1A, C, E, and G; Supplementary Fig. S3A and C). For example, only CTP levels increased in Vero cells, whereas both CTP and UTP were elevated in HCT116 cells. Minor increases in GTP were observed in A549 and U2OS cells (Fig. 1E; Supplementary Fig. S3A), suggesting cell-line-specific differences in rNuc salvage or metabolism.

Regarding dNTP levels, rNuc supplementation resulted in slight but statistically significant increases in dCTP in Vero, A549, HCT116, U2OS, and HT29 cells (Fig. 1D, F, and H; Supplementary Fig. S3B and D), but not in BJ cells (Fig. 1B). No significant changes were observed in other dNTPs. These observations are in line with previous results showing that treatment of cells with high concentrations (4000 μM or 8000 μM) of Cyd, Ado, or Guo markedly elevated CTP (10- to 15-fold), produced modest increases in ATP (∼1.5-fold) and GTP (∼2-fold), and had minimal effects on dNTP pools [21].

### dNuc treatment alters dNTP pools similarly to dThd alone

To compare the effect of rNuc and dNuc supplementation, we treated the same six cell lines with 50 μM and 250 μM dNuc mixes and measured rNTPs and dNTPs after 6 and 24 h. Across all cell lines, dTTP and dGTP levels increased, while dATP showed variable increases and dCTP levels declined (Fig. 1B, D, F, H; Supplementary Fig. S3B and D). These dNTP pool changes closely resembled those induced by dThd treatment alone, which is a well-established effect: dThd increases dTTP levels, which allosterically stimulates RNR to promote dGTP production while inhibiting dCTP synthesis. In turn, elevated dGTP levels promote dATP production through additional allosteric regulation [22, 23] (Supplementary Fig. S1). Notably, at high dThd concentrations (∼2000 μM), the resulting dCTP depletion leads to S-phase arrest, a principle exploited in the well-known double thymidine block method for cell cycle synchronization [24]. To confirm that the effects observed with the dNuc mixes were primarily driven by dThd, we treated several cell lines with dThd alone. The resulting dNTP pool alterations mirrored those seen with the complete dNuc mix (Fig. 1J).

Exogenous dThd did not affect rNTP pools (Fig. 1I), whereas supplementation of all four dNuc led to varying increases in GTP levels (Fig. 1A, C, E, and G; Supplementary Fig. S3A and C). This effect is likely caused by dGuo in the dNuc mix, which can be converted into guanine by purine nucleoside phosphorylase (PNP). Guanine is then converted to GMP by hypoxanthine-guanine phosphoribosyltransferase (HGPRT) and further phosphorylated to GTP. Previous studies have shown that guanine, even at concentrations as low as 25 μM, can increase GTP levels up to three-fold in A549 cells [25].

To confirm that dGuo metabolism was responsible for the increased GTP levels, we treated Vero cells with dNuc in combination with forodesine, a PNP inhibitor. This treatment completely abolished the GTP increase, confirming that dGuo was the source of the elevated GTP levels (Supplementary Fig. S4). Interestingly, Guo supplementation did not produce similar GTP increases, suggesting that dGuo is either more efficiently transported into cells or serves as a favored substrate for human PNP compared to Guo. Consistent with this, previous reports indicate that human PNP and its homologs from other organisms may preferentially process dGuo or its analogs over Guo [26, 27].

### The elevation of dNTP pools caused by EmbryoMax nucleosides is primarily attributed to the action of dThd

**Figure 2.**
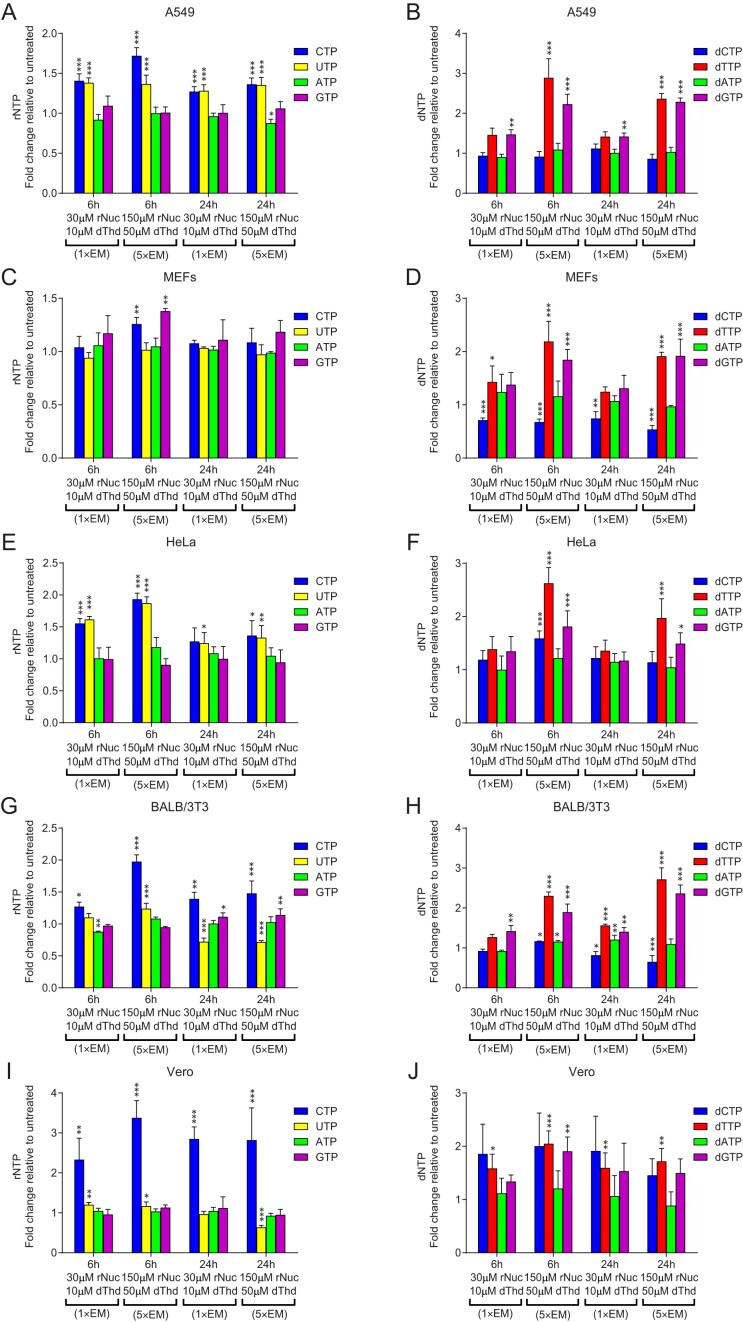
Changes in dNTP and rNTP pools in mammalian cells in response to EmbryoMax Nucleosides supplementation. (**A**, **C**, **E**, **G**, **I**) rNTP pools in A549, MEFs, HeLa, BALB/3T3, and Vero cells after 6 and 24 h of treatment with 1× EM Nuc (30 μM rNuc, 10 μM dThd) or 5× EM Nuc (150 μM rNuc, 50 μM dThd) relative to untreated controls. (**B**, **D**, **F**, **H**, **J**) dNTP pools in A549, MEF, HeLa, BALB/3T3, and Vero cells after 6 and 24 h of treatment with 1× EM Nuc (30 μM rNuc, 10 μM dThd) or 5× EM Nuc (150 μM rNuc, 50 μM dThd) relative to untreated controls. Error bars represent the SD from three independent experiments. Statistical significance: *P < .05, **P < .01, ***P < .001. No asterisk indicates *P* ≥ .05. Statistical significance was determined using two-way ANOVA followed by Bonferroni post hoc multiple comparisons.

We next analyzed dNTP and rNTP pools in five different cell lines supplemented with 1× and 5× EM Nuc, the nucleotide mix designed to increase dNTP pools and alleviate replication stress [9–15]. The changes in rNTP pools closely resembled those observed with rNuc alone (Fig. 2A, C, E, G, and I), while the changes in dNTP pools were similar to those seen with the dNuc mix or dThd alone: dTTP and dGTP increased (Fig. 2B, D, F, H, and J), suggesting that dThd in the EM Nuc mix was converted to dTTP, which allosterically stimulated RNR to produce dGTP (Supplementary Fig. S1).

However, while dCTP pools were generally decreased in cells treated with the dNuc mix or dThd (Fig. 1 and Supplementary Fig. S3), EM Nuc treatment produced a different outcome, where dCTP levels directly correlated with CTP levels. For example, in MEF cells, where no increase in CTP levels was observed (Fig. 2C), dCTP levels decreased (Fig. 2D). In A549, HeLa, and BALB/3T3 cells, where CTP levels increased up to ∼1.5-fold (Fig. 2A, E, and G), dCTP levels remained unchanged relative to the control (Fig. 2B, F, and H). In Vero cells, where CTP levels increased up to three-fold (Fig. 2I), dCTP levels also increased up to two-fold (Fig. 2J). These findings suggest that an increase in CTP levels, which are typically the lowest among all rNTPs in mammalian cells [16] (Supplementary Fig. S2), can stimulate dCTP production via RNR.

### Exogenous dThd alone accelerates replication fork speed, while rNuc do not

To investigate the impact of different nucleoside combinations on DNA replication fork speed, we performed DNA fiber assays in MEF, A549, and HCT116 cells. Prior to labeling nascent DNA with IdU and CldU, the cells were incubated for 48 h with 2× EM Nuc (60 μM rNuc and 20 μM dThd), 60 μM rNuc alone, or 20 μM dThd alone. Media was changed after 24 h with the corresponding nucleosides.

**Figure 3.**
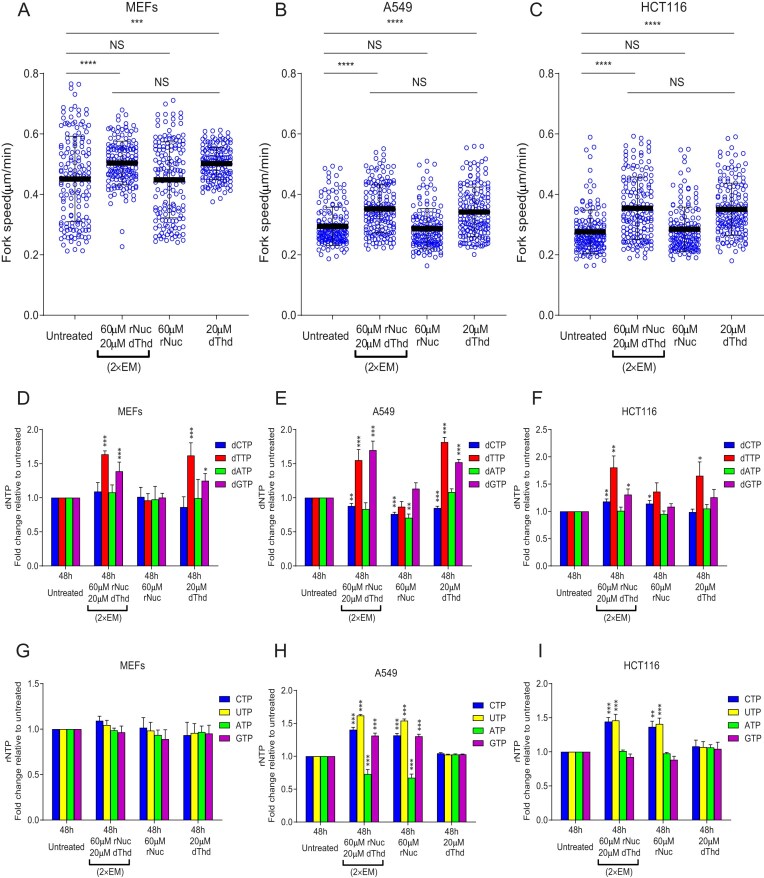
Impact of EmbryoMax Nucleosides, rNuc, or dThd supplementation on dNTP and rNTP pools and replication fork speed. (**A**–**C**) Replication fork speed in MEFs, A549, and HCT116 cells treated for 48 h with 2× EM Nuc (60 μM rNuc + 20 μM dThd), rNuc (60 μM), or dThd (20 μM). Asynchronously growing cells were labeled sequentially with IdUrd and CldUrd and analyzed using the DNA fiber assay. Representative results from two independent experiments are shown. NS = not significant (*P* ≥ .05), ***P < .001, ****P < .0001. Statistical significance was determined using the Mann–Whitney test. (**D**–**F**) Changes in dNTP pools relative to untreated controls. (**G**–**I**) Changes in rNTP pools relative to untreated controls. Error bars represent the SD from three independent experiments. Statistical significance: *P < .05, **P < .01, ***P < .001. No asterisk indicates *P* ≥ .05. Statistical significance was determined using one-way ANOVA followed by Bonferroni post hoc multiple comparisons.

We found that both EM Nuc and dThd alone significantly accelerated replication fork speed across all three cell lines, whereas rNuc had no effect (Fig. 3A–C). Analysis of dNTP and rNTP pools corroborated our previous findings: supplementation with 2× EM Nuc and 20 μM dThd increased dTTP and dGTP levels, while rNuc had no impact (Fig. 3D–I).

At the same time, cells treated with 20 μM dThd alone did not exhibit a pronounced decrease in dCTP levels (Fig. 3D–F), contrasting with the reduction observed at higher dThd concentrations (100 μM, Fig. 1J) or when dThd was included in the dNuc mix (50 μM and 250 μM, Fig. 1B, D, F, H and Supplementary Fig. S3B and D). This suggests that lower dThd concentrations may not elevate dTTP to a level sufficient to inhibit CDP reduction through allosteric regulation of RNR.

Interestingly, dCTP levels remained stable in MEF cells supplemented with 2× EM Nuc (Fig. 3D), in contrast to the decrease observed in Fig. 2D. We attribute this discrepancy to differences between MEF cells from different laboratories (Tsubouchi versus Chabes), possibly due to variations in nucleotide metabolism or salvage pathways.

### Inhibition of dUTPase leads to dUTP accumulation and slows replication fork speed, which is rescued by dThd

**Figure 4.**
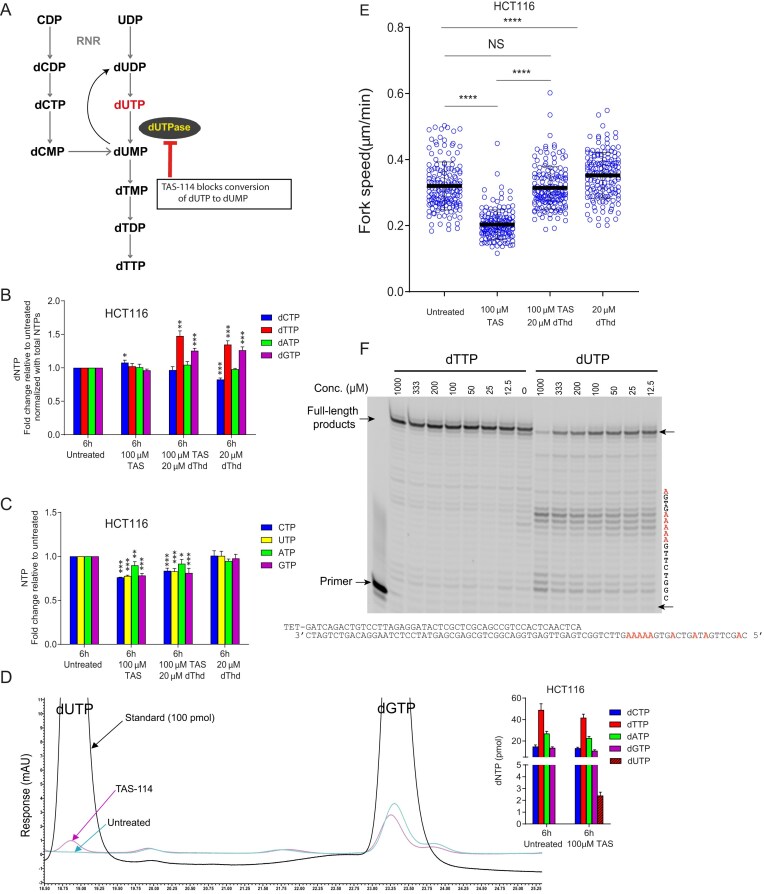
Impact of dUTPase inhibition on dNTP/rNTP pools and replication fork speed in HCT116 cells. (**A**) Schematic representation of the metabolic pathway leading to dTTP production. (**B**) Changes in dNTP pools relative to untreated controls. Error bars represent SD from three independent experiments. Statistical significance: *P < .05, **P < .01, ***P < .001. No asterisk indicates *P* ≥ .05. Statistical significance was determined using one-way ANOVA followed by Bonferroni post hoc multiple comparisons. (**C**) Changes in rNTP pools relative to untreated controls. Error bars represent SD from three independent experiments. Statistical significance: *P < .05, **P < .01, ***P < .001. No asterisk indicates *P* ≥ .05. Statistical significance was determined using one-way ANOVA followed by Bonferroni post hoc multiple comparisons. (**D**) HPLC chromatogram showing dUTP and dGTP standard peaks, as well as dUTP and dGTP peaks in HCT116 cells treated with 100 μM TAS-114 and untreated controls. The inset presents absolute dNTP amounts, including dUTP, in untreated and TAS-114–treated cells. (**E**) Replication fork speed in HCT116 cells treated for 6 h with 100 μM TAS-114, 20 μM dThd, or their combination. Asynchronously growing cells were labeled sequentially with IdUrd and CldUrd and analyzed using the DNA fiber assay. Representative results from two independent experiments are shown. Values represent the mean ± SD. NS = not significant (*P* ≥ .05), ****P < .0001. Statistical significance was determined using the Mann–Whitney test. (**F**) Effect of dTTP or dUTP on DNA synthesis by human POLE1 in the presence of 8 μM dTTP, 4 μM dCTP, 4 μM dATP, and 2 μM dGTP. The template sequence is shown next to the gel, while the primer and template are depicted below the gel.

To further investigate why dTTP accelerates replication fork speed, we examined whether dUTP negatively affects DNA synthesis, as dUTP and dTTP share metabolic pathways and compete for incorporation into DNA. dUTP can be generated through two pathways (Fig. 4A): (i) RNR reduces UDP to dUDP, which is then phosphorylated to dUTP and (ii) RNR reduces CDP to dCDP, which is converted to dCMP, deaminated to dUMP by dCMP deaminase, phosphorylated to dUDP, and finally to dUTP. Incorporation of dUTP into DNA is undesirable because uracil is recognized as a lesion, triggering base excision repair and potentially leading to breaks or stalled forks if repair coincides with ongoing replication.

To prevent dUTP incorporation, mammalian cells express dUTPase (also known as DUT), a highly active enzyme that rapidly hydrolyzes dUTP to dUMP. To determine whether elevated dUTP alone hinders replication fork speed, we treated HCT116 cells for 6 h with TAS-114, a specific dUTPase inhibitor that increases uracil incorporation into genomic DNA [28, 29] (Fig. 4A). Measurements of dNTP and rNTP pools showed that TAS-114 treatment did not substantially alter dNTP levels and caused only minor changes in rNTP levels (Fig. 4B and C), indicating that dUTPase activity is not essential for dTTP production. This supports earlier findings that the primary pathway for dTTP synthesis in mammalian cells occurs via CDP reduction rather than UDP reduction [30, 31]. However, in TAS-114–treated cells, dUTP became readily detectable, reaching up to 20%–25% of dCTP and dGTP levels (Fig. 4D).

After 6 h of TAS-114 treatment, replication fork speed was markedly reduced (Fig. 4E), suggesting that accumulated dUTP impaired normal replication. Strikingly, co-treatment with 20 μM dThd restored replication fork speed, reversing the effect of TAS-114. This dual treatment led to a ∼1.25-fold increase in both dTTP and dGTP pools, matching the increases observed with dThd alone (Fig. 4B). These findings suggest that increasing dTTP levels through exogenous dThd can counteract the negative effect of excess dUTP on fork progression.

To further explore how dUTP might affect DNA synthesis, we performed *in vitro* primer extension assays using the catalytic subunit of human Pol ϵ. When dUTP was included in the nucleotide pool, reproducible pausing occurred at adenine sites in the template strand (Fig. 4F). Although all primers eventually extended to full length under these conditions, the appearance of stalled or intermediate products suggests that incorporation of dUTP, unlike canonical nucleotides, transiently impedes Pol ϵ-mediated elongation. Importantly, our assay design reflects a short-term (6 h) increase in dUTP in cells and specifically addresses whether incorporation of dUTP itself can perturb synthesis, in contrast to a recent study that examined polymerase bypass of uracil in the template DNA [29]. While our system does not capture the complete holoenzyme or physiological nucleotide ratios, it provides evidence that dUTP incorporation can itself represent a barrier to efficient DNA replication.

## Discussion

rNuc, either alone or in combination with dThd (such as in EmbryoMax Nucleosides, EM Nuc), are widely used to improve replication dynamics and are commonly described as relieving replication stress in the literature. They are also a standard component of media for growing embryonic and induced pluripotent stem cells [5–15]. This study separates the effects of rNuc from those of dThd. rNuc mainly change rNTPs (CTP and UTP) and have little effect on dNTPs, while dThd increases dTTP and, through RNR allostery, also raises dGTP. These changes are linked to faster replication forks. EM Nuc behaves likewise because of its dThd component. Elevated dTTP also counterbalances dUTP-linked inhibition of replication, supporting a mechanistic basis for dThd-mediated stress relief.

### Impact of rNuc supplementation on nucleotide pools

Supplementation with rNuc led to elevated CTP and UTP pools across different cell lines without significantly altering dNTP pools, apart from minor but statistically significant increases in dCTP levels in some of the tested cell lines (Fig. 1). We propose that the increase in dCTP pools is driven by the elevation of CDP levels. Among rNTPs, CTP is present at the lowest intracellular concentration in mammalian cells (ATP ∼3200 μM, GTP ∼470 μM, UTP ∼570 μM, CTP ∼280 μM) [16, 18] (Supplementary Fig. S2). Since rNMP, rNDP, and rNTP pools generally follow a 1:10:100 ratio [32], the CDP concentration is presumably the lowest among the four RNR substrates. Consequently, it may be a limiting factor in dCDP production and could be most easily influenced by exogenous rNuc supplementation.

The lack of a significant increase in dNTP pools following rNuc treatment at 50 μM (Fig. 1B, D, F, H; Supplementary Fig. S3B and D) raises questions about how rNuc alleviate replication stress, as reported in previous studies [5–8]. One possible explanation is that the cells used in those studies had reduced rNTP pools, which in turn contributed to lower dNTP pools. For instance, Bester *et al.* reported that cells with decreased dNTP pools, caused by the expression of HPV-16 E6 and E7 proteins, also exhibited reduced rNTP pools [5]. Thus, in such conditions, rNuc supplementation may alleviate replication stress by replenishing rNTP (and rNDP) pools, indirectly supporting dNTP synthesis and DNA replication. Given that only CTP and UTP levels increased in response to rNuc treatment (Fig. 1), future studies should examine whether a complete set of rNuc is necessary for alleviating replication stress in such contexts.

### Impact of dNuc and dThd supplementation on nucleotide pools

Unlike rNuc, supplementation with either dNuc or dThd alone had a pronounced effect on dNTP pools. Both treatments led to increased levels of dTTP and dGTP across all cell lines, with varying degrees of dATP elevation and dCTP reduction depending on the cell line. These changes are primarily driven by the dThd-mediated elevation of dTTP levels, which allosterically stimulates RNR to produce more dGTP (Supplementary Fig. S1).

Interestingly, despite the presence of both dCyd and dThd in the dNuc mix, dCTP levels were reduced in dNuc-treated cells. This contradicts earlier reports suggesting that dCyd supplementation can restore dCTP pools diminished by dThd treatment [22, 33]. However, other studies show that exogenous dCyd is not efficiently converted to dCTP in actively dividing cells and is therefore less readily incorporated into replicating DNA. In contrast, dCTP derived from exogenous Cyd appears to be preferentially utilized for DNA synthesis [34–36]. These latter observations are consistent with our results, as we did not observe a restoration of dCTP levels following dCyd and dThd supplementation in the dNuc mix (Fig. 1), whereas the combination of Cyd and dThd in EM Nuc did restore dCTP levels in some cell lines (Fig. 2). Differences among studies may stem from variation in salvage pathway efficiency, including differences in transport and phosphorylation efficiency of Cyd and dCyd, which can vary between cell lines.

### Impact of rNuc and dThd supplementation on replication fork speed

Changes in dNTP levels in cells treated with 60 μM rNuc plus 20 μM dThd (equivalent to the 2× EM Nuc mix) were similar to those observed in cells treated with 20 μM dThd alone, while treatment with 60 μM rNuc alone produced no significant or only minor effects on dNTP pools (Fig. 3D–F). Consistently, replication fork speed was similarly accelerated in cells treated with 60 μM rNuc plus 20 μM dThd compared to dThd alone, whereas no acceleration was observed with rNuc alone. These findings strongly suggest that dThd is the primary driver of the fork-accelerating effects of EM Nuc, although a minor contribution from Cyd—compensating for potential dCTP deficits—cannot be ruled out in certain cell lines.

Several non-mutually exclusive mechanisms may explain how dThd treatment accelerates replication. First, dTTP and dGTP—the two dNTPs most significantly elevated by dThd—may be naturally limiting for replication fork speed. In budding yeast, physiological dNTP levels are known to restrict replication fork progression [37, 38], and a similar constraint may apply in mammalian cells.

Notably, dGTP concentration is typically the lowest of the four dNTPs in proliferating mammalian cells [16], and even modest increases could directly support faster fork progression. Thus, the acceleration we observe may result from combined effects of elevated dTTP and dGTP rather than a single limiting factor.

Second, we show that dUTP accumulation, caused by dUTPase inhibition via TAS-114 [28, 29], significantly slows replication fork progression, an effect reversed by exogenous dThd (Fig. 4). Our biochemical assays suggest that dUTP can impede Pol ϵ activity by triggering pausing events during incorporation opposite template adenines. These experiments were performed with the catalytic subunit alone, under conditions with excess dUTP relative to dTTP, and thus do not reflect the full physiological context of the holoenzyme, PCNA, or chromatin. Rather, they demonstrate that dUTP incorporation can in principle perturb DNA synthesis. Nevertheless, the reproducible accumulation of stalled products indicates that dUTP incorporation itself can pose a barrier to DNA synthesis.

This finding is complementary to the recent work of Saxena and colleagues [29]. In their study, a purified human Pol ϵ catalytic fragment lacking exonuclease activity (Pol ϵ exo–) was tested on synthetic DNA templates containing one or two consecutive uracils. They observed pausing on uracil-containing templates, particularly when two consecutive uracils were present, whereas our assays addressed competition between dUTP and dTTP at the point of incorporation. Importantly, their experimental design reflects prolonged (24 h) elevation of dUTP in cells, when uracil bases have already been incorporated into genomic DNA, and focuses exclusively on bypass of these lesions. In contrast, our assays mimic short-term (6 h) increases in dUTP and test whether incorporation of dUTP itself perturbs Pol ϵ-dependent DNA synthesis. Together, these complementary approaches suggest that elevated dUTP may impair replication at two distinct levels: (i) during incorporation opposite adenines and (ii) during bypass of uracil residues once incorporated. Both mechanisms likely contribute to fork slowdown under conditions of an altered dUTP/dTTP balance.

Other biochemical studies reported that proofreading-proficient bacterial and yeast polymerases do not necessarily stall at single uracils in template DNA [39], suggesting that polymerase responses may depend on the enzyme system and the number of uracils present. Thus, uracil may impair replication both through inefficient incorporation events and by blocking elongation when present in the template strand. Further studies are needed to disentangle the relative contribution of these mechanisms. Additionally, thymidine has been reported to rescue ATR kinase inhibitor-induced uracil accumulation in genomic DNA, although the origin of uracil in that context was unclear, since dUTP levels were paradoxically decreased under ATR inhibition [40].

Interestingly, dTTP levels are consistently the highest among all dNTPs in actively dividing eukaryotic cells [16]. While the precise reason for this remains unknown, one possibility is that elevated dTTP serves as a protective mechanism to outcompete residual dUTP, thereby reducing the risk of uracil misincorporation and the associated DNA repair burden. Given that dUTP is an unavoidable byproduct of pyrimidine metabolism, maintaining high dTTP levels may be a critical strategy for preserving genomic integrity. In addition, elevated dTTP may also compete with rUTP incorporation, since ribonucleotides embedded in DNA can be bypassed by mutagenic translesion synthesis polymerases in yeast [41]. Accordingly, high dTTP may function as a general protective mechanism against both dUTP- and rUTP-mediated genome instability.

Beyond direct dNTP elevation, the effects of rNuc or dNuc supplementation on replication dynamics may involve indirect mechanisms, such as signaling through nucleoside receptors [42, 43]. Our findings refine the understanding of nucleoside supplementation strategies and provide a mechanistic basis for their effects on nucleotide metabolism and replication fork progression, with potential implications for optimizing cell culture conditions and therapeutic interventions targeting nucleotide metabolism.

## Supplementary Material

gkaf1035_Supplemental_File

## Data Availability

The data underlying this article are available in the article and in its online supplementary material.

## Notes

Present address: Department of Applied Life Sciences, Faculty of Agriculture, Shizuoka University, 836 Oya, Suruga-ku, Shizuoka422-8529, Japan
