# Correction to ‘Decoding nucleoside supplementation: how thymidine outperforms ribonucleosides in accelerating mammalian replication forks’

**DOI:** 10.1093/nar/gkag532

**Published:** 2026-05-20

This is a correction to: Praveen Pandey, Kiminori Kurashima, Göran Bylund, Erik Johansson, Tomomi Tsubouchi, Andrei Chabes, Decoding nucleoside supplementation: how thymidine outperforms ribonucleosides in accelerating mammalian replication forks, *Nucleic Acids Research*, Volume 53, Issue 19, 28 October 2025, gkaf1035, https://doi.org/10.1093/nar/gkaf1035

In the originally published manuscript, the ‘**Introduction**’ section contained an error introduced during production. The relevant sentence has been corrected to read: “Notably, this formulation also includes one deoxyribonucleoside (dNuc), 1 mM thymidine (dThd)” instead of: “Notably, this formulation also includes one dNuc and 1 mM thymidine (dThd)”.

The published article has been updated.

