# Supplementary material for: Decoding nucleoside supplementation: how thymidine outperforms ribonucleosides in accelerating mammalian replication forks
Source: Nucleic Acids Res. 2025 Oct 14;53(19):gkaf1035. doi: 10.1093/nar/gkaf1035 (PMC12526065; doi:10.1093/nar/gkaf1035)
Supplement: gkaf1035_Supplemental_File [file gkaf1035_supplemental_file.pdf]

Supplementary Figure 1

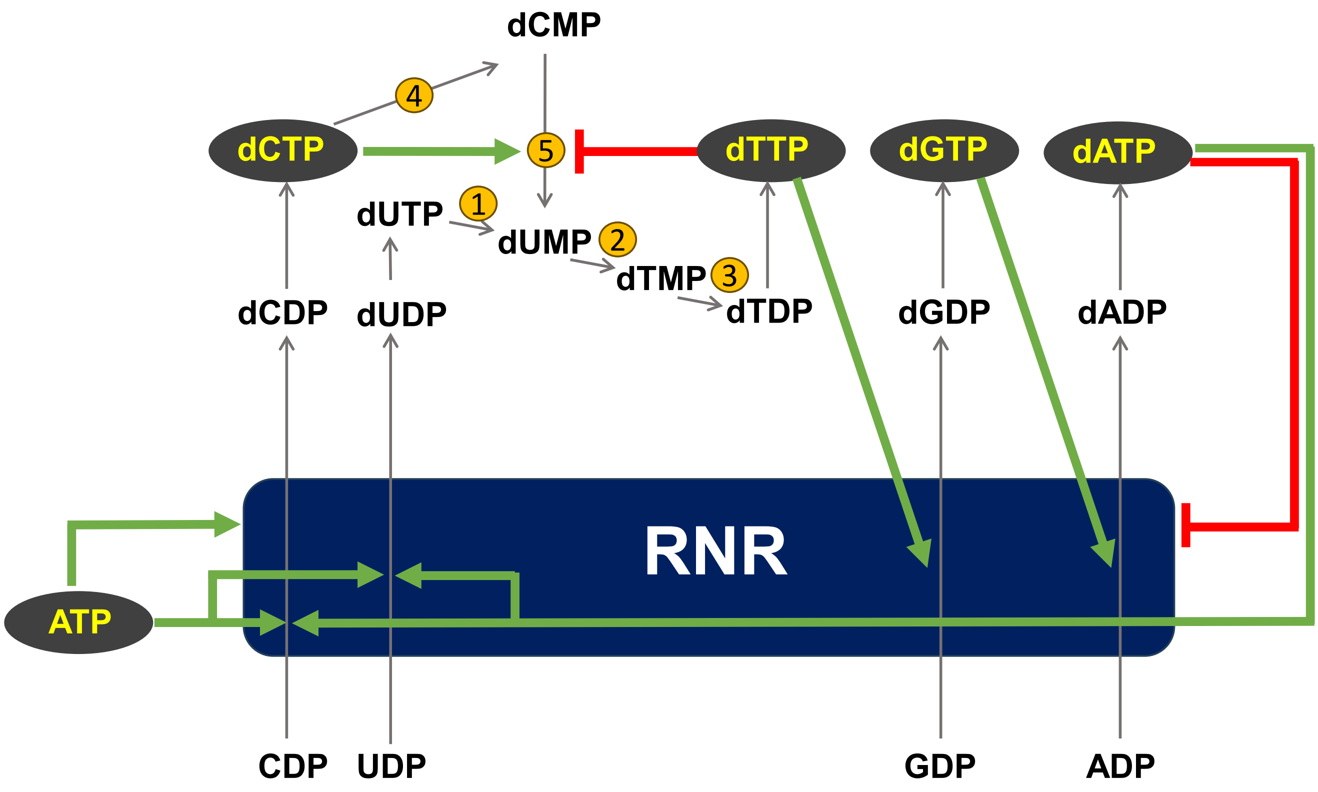

Supplementary Figure 1. Schematic overview of *de novo* dNTP synthesis in mammalian cells.

RNR converts rNDPs to dNDPs, which are then phosphorylated to dNTPs by nucleoside diphosphate kinases. Key enzymes include: 1. dUTPase, 2. Thymidylate synthase, 3. Thymidylate kinase, 4. dCTPase, 5. dCMP deaminase. Green arrows indicate allosteric stimulation, while red arrows denote allosteric inhibition. Stimulation of one RNR activity inhibits its other activities. For example, dTTP stimulates GDP reduction and inhibits CDP, UDP, and ADP reduction. ATP stimulates both overall RNR activity and the reduction of CDP and UDP. dATP, at low concentrations, stimulates CDP and UDP reduction, while at high concentrations, it inhibits overall RNR activity.

Supplementary Figure 2

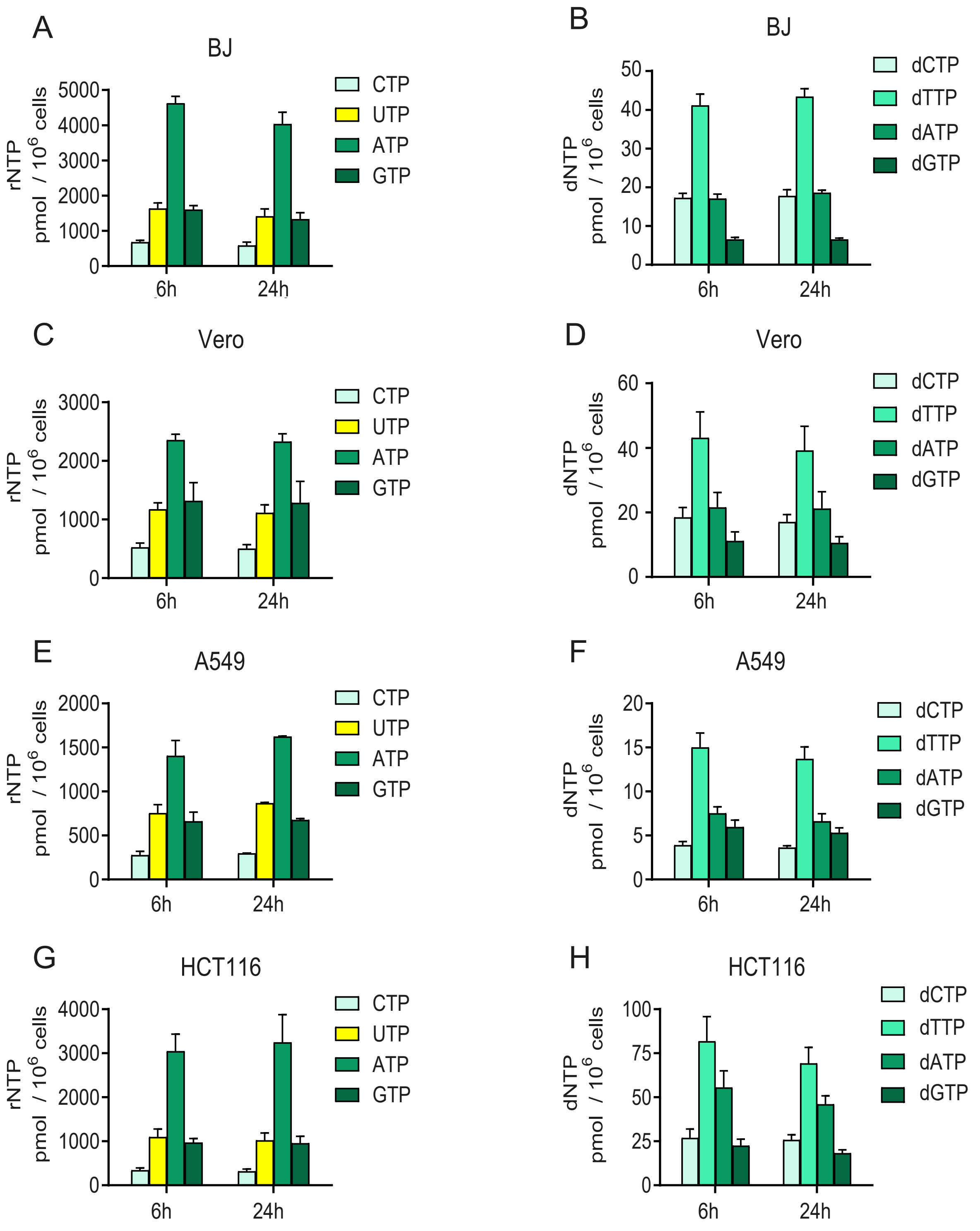

Supplementary Figure 2. rNTP and dNTP pool balances in proliferating mammalian cells

(A, C, E, G) rNTP pool balances in untreated BJ, Vero, A549, and HCT116 cells 6 and 24 hours after plating.

(B, D, F, H) dNTP pool balances in untreated BJ, Vero, A549, and HCT116 cells 6 and 24 hours after plating.

Error bars represent the SD from three independent experiments.

Supplementary Figure 3

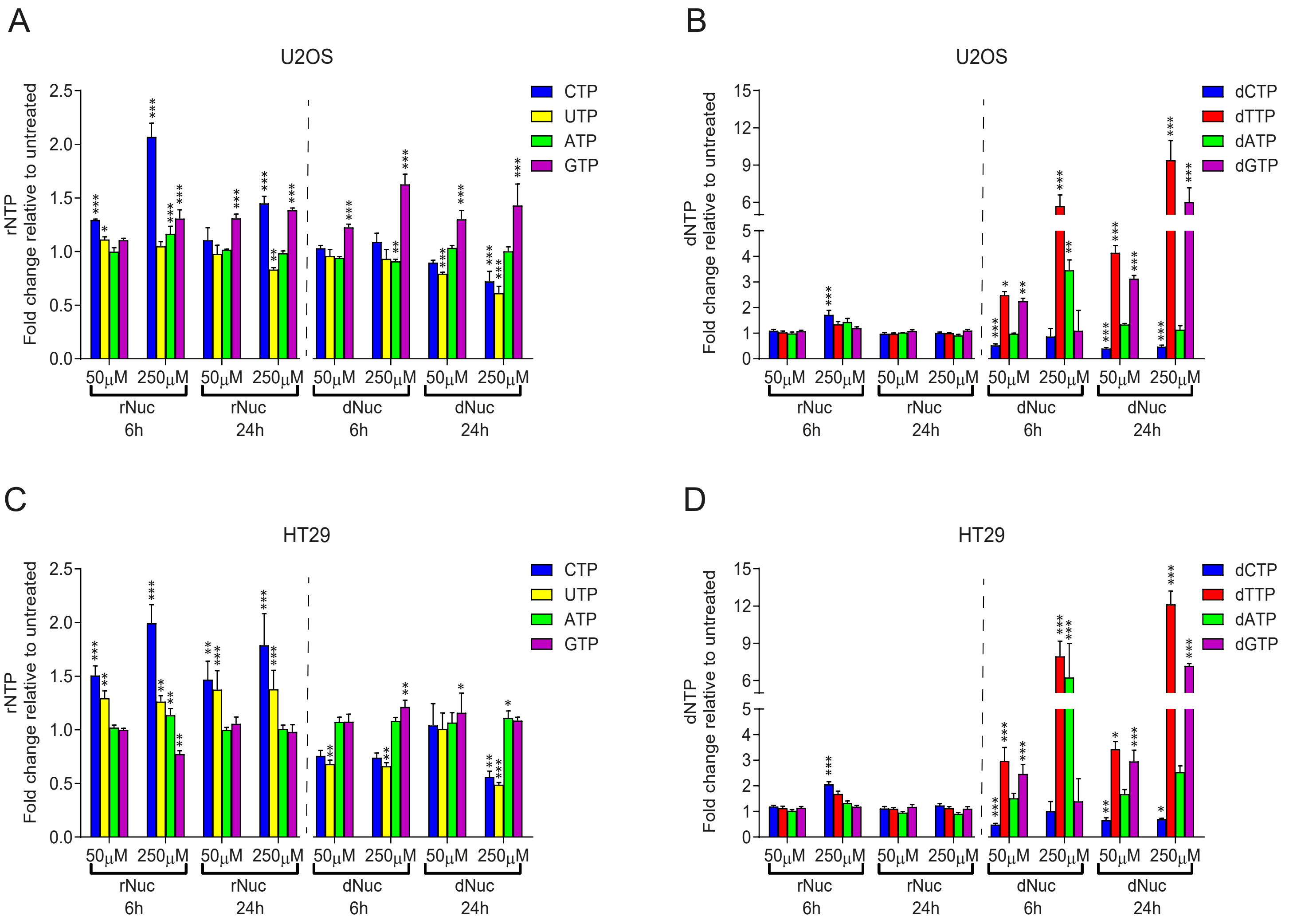

Supplementary Figure 3. Changes in dNTP and rNTP pools in mammalian cells in response to rNuc or dNuc supplementation.

(A and C) rNTP pools in U2OS and HT29 cells after 6 and 24 h of treatment with rNuc (50 µM or 250 µM) or dNuc (50 µM or 250 µM) relative to untreated controls.

(B and D) dNTP pools in U2OS and HT29 cells after 6 and 24 h of treatment with rNuc (50 µM or 250 µM) or dNuc (50 µM or 250 µM) relative to untreated controls.

Error bars represent the SD from three independent experiments. Statistical significance: *p<0.05, **p<0.01, ***p<0.001. No asterisk indicates p ≥ 0.05. Statistical significance was determined using tw way ANOVA followed by Bonferroni post hoc multiple comparisons.

Supplementary Figure 4

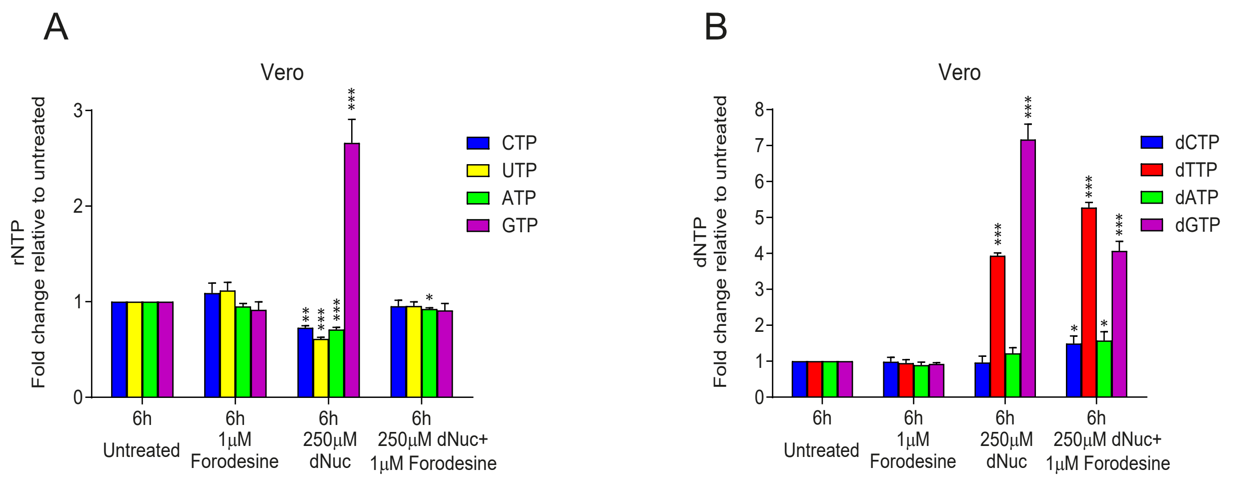

Supplementary Figure 4. Treatment with Forodesine abolishes the GTP pool increase caused by treatment with dNuc.

(A) dNTP pools in Vero cells after 6 h of treatment with dNuc, dNuc + Forodesine mix, or Forodesine alone, relative to untreated controls.

(B) rNTP pools in Vero cells after 6 h of treatment with dNuc, dNuc + Forodesine mix, or Forodesine alone, relative to untreated controls.

Error bars represent the SD from three independent experiments. Statistical significance: *$p<0.05$, **$p<0.01$, ***$p<0.001$. No asterisk indicates $p \geq 0.05$. Statistical significance was determined using one-way ANOVA followed by Bonferroni post hoc multiple comparisons.

Supplementary Table S1. Acronyms for nucleosides and nucleotides used in this study

Acronym	Full name	Description
rNuc	Ribonucleoside	Ado, Cyd, Guo, Urd
dNuc	Deoxyribonucleoside	dAdo, dCyd, dGuo, dThd
EM Nuc	EmbryoMax® Nucleosides	Commercial mix: 3 mM of each rNuc + 1 mM dThd
Ado	Adenosine	Ribonucleoside
Urd	Uridine	Ribonucleoside
Cyd	Cytidine	Ribonucleoside
Guo	Guanosine	Ribonucleoside
dAdo	Deoxyadenosine	Deoxyribonucleoside
dCyd	Deoxycytidine	Deoxyribonucleoside
dGuo	Deoxyguanosine	Deoxyribonucleoside
dThd (Thd, dT)	Deoxythymidine (thymidine)	Deoxyribonucleoside
ATP	Adenosine triphosphate	Ribonucleoside triphosphate, rNTP
GTP	Guanosine triphosphate	Ribonucleoside triphosphate, rNTP
CTP	Cytidine triphosphate	Ribonucleoside triphosphate, rNTP
UTP	Uridine triphosphate	Ribonucleoside triphosphate, rNTP
dATP	Deoxyadenosine triphosphate	Deoxyribonucleoside triphosphate, dNTP
dGTP	Deoxyguanosine triphosphate	Deoxyribonucleoside triphosphate, dNTP
dCTP	Deoxycytidine triphosphate	Deoxyribonucleoside triphosphate, dNTP
dTTP	Deoxythymidine triphosphate	Deoxyribonucleoside triphosphate, dNTP
dUTP	Deoxyuridine triphosphate	Non-canonical dNTP
